# Primary Cutaneous Nocardiosis of the Scrotum Following Presurgical Inguinal Shaving

**DOI:** 10.4269/ajtmh.25-0285

**Published:** 2025-08-19

**Authors:** Nathalia Chebli-de-Abreu, Júlia Amélia Ricci, Débora Dumont Cruz Nunes

**Affiliations:** ^1^ Division of Dermatology, Federal University of Juiz de Fora, Juiz de Fora, Brazil;; ^2^ Departament of Dermatology, Eduardo de Menezes Hospital, Belo Horizonte, Brazil

**Figure 1.**
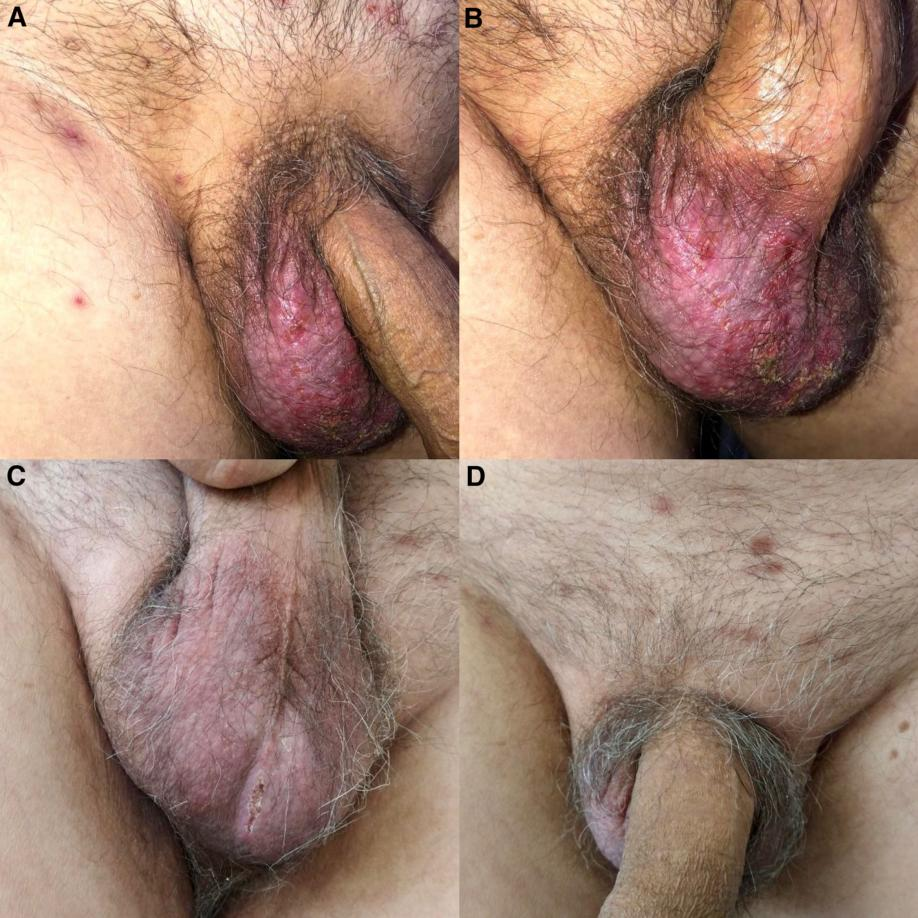
(**A** and **B**) Erythematous scrotal plaque with crusts, exulcerations, and satellite papulopustules. (**C** and **D**) Clinical improvement after 1 month of treatment.

**Figure 2.**
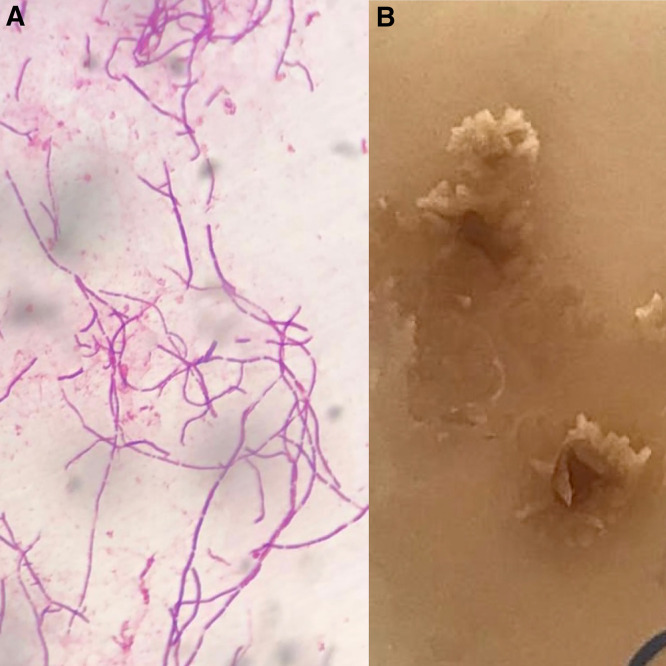
(**A**) Gram stain showing thin, branching, Gram-positive filaments consistent with *Nocardia* spp. (**B**) Chalky-white colonies of *Nocardia* spp. on Sabouraud dextrose agar at 25°C after 10 days of incubation.

A 69-year-old man presented with a 3-week history of pruritic scrotal lesions after presurgical hair removal for a coronary angioplasty. One week before presentation, he had undergone inguinal shaving with a disposable razor in a hospital surgical setting. Physical examination revealed an erythematous plaque with crusts and exulcerations on the right scrotum, accompanied by satellite papules and pustules (Figure 1). A direct smear from a pustule revealed numerous thin, clustered, and branching Gram-positive filaments (Figure 2A), which were partially acid-fast with Kinyoun stain. A culture of skin swabs on Sabouraud dextrose agar at 25°C yielded *Nocardia* spp., which formed small, chalky-white, heaped, wrinkled, or verrucous colonies with a distinctive odor reminiscent of freshly turned soil (Figure 2B). Lymphatic and systemic involvement were excluded by imaging.

A diagnosis of superficial cutaneous nocardiosis, likely due to direct inoculation, was confirmed. Oral trimethoprim (160 mg) and sulfamethoxazole (800 mg), taken three times daily, were initiated. Significant improvement was noted after 1 month (Figure 1C and D), and therapy was continued for an additional 3 months until complete resolution.

*Nocardiosis* is an uncommon opportunistic infection caused by aerobic, Gram-positive bacilli of the *Nocardia* genus. It mimics a variety of dermatoses, often delaying diagnosis despite the availability of straightforward microbiologic methods.^1^^–^^3^ A high index of suspicion, clinical awareness, and timely microbiologic evaluation are critical for accurate diagnosis. In this case, an unusual route of inoculation through presurgical shaving is illustrated, highlighting the importance of infection control practices and the recognition of atypical cutaneous infections.
